# Using Commercial Activity Monitors to Measure Gait in Patients with Suspected iNPH: Implications for Ambulatory Monitoring

**DOI:** 10.7759/cureus.382

**Published:** 2015-11-17

**Authors:** Shiv Gaglani, Jessica Moore, M Ryan Haynes, Jamie B Hoffberger, Daniele Rigamonti

**Affiliations:** 1 Department of Neurosurgery, The Johns Hopkins University School of Medicine; 2 Department of Radiation Oncology, The Johns Hopkins University School of Medicine

**Keywords:** idiopathic normal pressure hydrocephalus, inph, activity monitor

## Abstract

Objectives: This study seeks to validate the use of activity monitors to detect and record gait abnormalities, potentially identifying patients with idiopathic normal pressure hydrocephalus (iNPH) prior to the onset of cognitive or urinary symptoms.

Methods: This study compared the step counts of four common activity monitors (Omron Step Counter HJ-113, New Lifestyles 2000, Nike Fuelband, and Fitbit Ultra) to an observed step count in 17 patients with confirmed iNPH.

Results: Of the four devices, the Fitbit Ultra (Fitbit, Inc., San Francisco, CA) provided the most accurate step count. The correlation with the observed step count was significantly higher (p<0.009) for the Fitbit Ultra than for any of the other three devices.

Conclusions: These preliminary findings suggest that existing activity monitors have variable efficacy in the iNPH patient population and that the MEMS tri-axial accelerometer and algorithm of the Fitbit Ultra provides the most accurate gait measurements of the four devices tested.

## Introduction

Patients with idiopathic normal pressure hydrocephalus (iNPH) commonly present with a triad of symptoms consisting of gait, cognitive, and/or urinary difficulties [1-2]. While patients can often retrospectively identify a gradual loss of functionality, current clinical monitoring cannot detect these subtle changes. Activity monitors present an opportunity for monitoring gait abnormalities in iNPH patients. These devices have been studied in the monitoring of gait abnormalities in patients with chronic diseases, including cerebral palsy, multiple sclerosis, Parkinson’s disease, traumatic brain injury, stroke, rheumatoid arthritis, and osteoarthritis [3-9]. While these studies demonstrate the potential of activity monitors in clinical use and the need for ambulatory monitoring in many chronic conditions, they reported highly variable reliabilities in the gait monitoring of the activity monitors, concluding that the selection of an activity monitor must be application- and condition-specific [10-11]. In iNPH, monitoring the progression of symptoms in an ambulatory setting would require models of normal gait and the progression of iNPH gait changes.

The objectives of this study were to determine whether an activity monitoring device could accurately record gait in iNPH patients and, if so, which devices were most reliable in their step counts.

## Materials and methods

Seventeen patients with suspected iNPH based on cognitive and balance evaluations and MRI were enrolled to test the activity monitors. Activity monitors varied in the type of sensor, mounting location, and cost. The four monitors used were the Omron Step Counter HJ-113 (Omron Corp., Kyoto, Japan), New Lifestyles 2000 (NEW LIFESTYLES, Inc., Lee's Summit, MO), Nike Fuelband (Nike, Inc., Beaverton, OR), and Fitbit Ultra (Fitbit, Inc., San Francisco, CA). Patients wore the activity monitors on their right hip during two trials of a 10-meter walk. The trials were recorded on video. The step count was measured both by the pedometers and by two observers. The step counts measured by the observers were assessed for any differences and were reconciled by watching the video tape in the event of a disagreement. The correlation between the two step count values was calculated using linear regression. The correlation between the step count values of each device were calculated using bivariate correlation coefficient methods [12]. Step count variability between manual observation and device measurement was assessed using concordance correlation coefficients. Differences between measurements were plotted against average measurements using Bland-Altman plots. 

Approval for this study was received from the Johns Hopkins Institutional Review Board (approval #NA_00077065). Informed consent was obtained from each participant.

## Results

**Figure 1:**
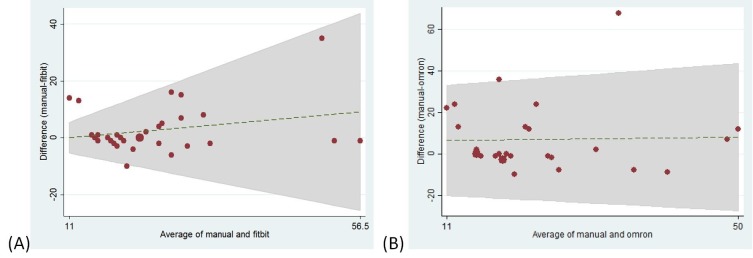
Bland-Altman Plot of Manual vs. (A) Fitbit Step Count or (B) Omron Step Count

The 17 patients ranged in age from 31 to 83 years, with a mean age of 69.7 years. Correlations between the step count values of the pedometer and observer were poor (R^2^<0.15) for the Omron Step Counter JH-113, New Lifestyles 2000, and Nike Fuelband. Correlation of the step count values was significantly higher for the Fitbit Ultra (R^2^=0.57). The correlation of the observed step count value was significantly better with the step count of the Fitbit Ultra than with the Omron Step Counter HJ-113 (p<0.001), the New Lifestyles 2000 (p<0.001), and the Nike Fuelband (p=0.009). Concordance correlation coefficients (CCC) between the device and manual step counts showed significant correlation only for the Fitbit Ultra (Table 1). The Bland-Altman plot of Fitbit versus manual step count showed a trend towards larger differences at higher step counts, but agreement at lower step counts (Figure 1). Similar plots for Omron, New Lifestyles, and Nike Fuelband activity monitors (not shown) showed relatively constant levels of difference regardless of step count with no areas of precise agreement.

**Table 1:** Concordance Correlation Coefficients for Activity Monitor and Manual Step Counts

Device	Rho	95% CI	Count Difference, Manual–Device (95% CI)
Fitbit Ultra	0.72	0.56, 0.89	2.74 (-14.0, 19.5)
Omron	0.19	-0.14, 0.53	7.0 (-25.8, 39.7)
New Lifestyles	-0.27	-0.56, 0.02	8.4 (-30.5, 47.4)
Nike Fuelband	-0.08	-0.29, 0.12	14.9 (-7.3, 37.1)

## Discussion

While the accuracy of different activity monitors has been explored for a variety of neurological diseases, the use of activity monitors in the iNPH population has not been previously explored. Preliminary findings suggest that commercial activity monitors have a variable efficacy in the iNPH patient population. Of the four devices tested, the MEMS triaxial accelerometer and algorithm of the Fitbit Ultra was the most accurate at recording step counts in patients with iNPH. The step count measured by the Fitbit Ultra had a significantly higher correlation with the observed step count than any of the other three devices tested, as well as a significant, positive concordance correlation coefficient. However, the accuracy of the Fitbit Ultra was still relatively low, suggesting that the existing algorithms for accelerometers may be insufficient to measure the gait changes in iNPH. Further testing is required to assess other gait characteristics, such as step length, step height, and balance, as markers of neurological deterioration. Given the chronic nature of iNPH and the benefit from early detection of symptom progression, though, we believe that activity monitors still possess great potential to improve the quality of life of patients with iNPH.

Limitations of this study include the small study population and the limited number of devices tested. Furthermore, while this study provides preliminary data about the accuracy of different activity monitors, further testing is required to determine the accuracy of monitors in detecting gait changes over time, as this would determine the clinical utility of such devices. In addition, this study was unable to account for the degree of abnormality in a particular patient’s gait. Therefore, the accuracy of different monitors at different severities of gait abnormalities could not be assessed. 

Despite these limitations, this data provides a first step into applying activity monitors to the management of this chronic disease. The variable accuracy of activity monitors in the iNPH population is in keeping with studies of activity monitor accuracy in patients with other neurological diseases. These findings support the need for further testing of specific devices prior to their integration into a chronic disease care model.

The utility of activity monitors in iNPH monitoring and management depends on whether clinically relevant data may be collected. This will require longer-term monitoring of iNPH patients in the ambulatory setting, including monitoring of patients one-week pre- and one-week post-lumbar puncture. If clinically relevant data can be collected, ambulatory monitoring may greatly benefit patients with iNPH and other chronic disorders in the future by offering healthcare providers information about the daily function and trends in symptoms.

## Conclusions

As activity monitors become increasingly common, healthcare providers should seize the opportunity to engage patients in their own health maintenance [13]. This is particularly true for patients with chronic disease, about whom information about the daily function and long-term changes may be difficult to obtain. In this preliminary study, the Fitbit Ultra activity monitor proved to be the most accurate in monitoring the steps of patients with idiopathic normal pressure hydrocephalus, although even the accuracy of the Fitbit was limited (R^2^=0.56, CCC=0.76). Further studies are needed to illuminate whether activity monitors can identify early changes in patients with iNPH, allowing them to obtain treatment before significant symptom progression and impairment.
